# Heat-tolerant rice cultivars retain grain appearance quality under free-air CO_2_ enrichment

**DOI:** 10.1186/s12284-014-0006-5

**Published:** 2014-05-21

**Authors:** Yasuhiro Usui, Hidemitsu Sakai, Takeshi Tokida, Hirofumi Nakamura, Hiroshi Nakagawa, Toshihiro Hasegawa

**Affiliations:** 1 National Institute for Agro-Environmental Sciences, 3-1-3 Kannondai, Tsukuba 305-8604, Ibaraki, Japan; 2 Taiyo Keiki Co. Ltd, 1-12-3, Nakajujo, Kita-ku 114-0032, Tokyo, Japan; 3 NARO Agricultural Research Center, 3-1-1 Kannondai, Tsukuba 305-8666, Ibaraki, Japan

**Keywords:** Chalky grains, Climate change, FACE (free-air CO2 enrichment), Heat-tolerant cultivars, Oryza sativa

## Abstract

**Background:**

Heat-tolerant rice cultivars have been developed as a countermeasure to poor grain appearance quality under high temperatures. Recent studies showed that elevated CO_2_ concentrations (E-[CO_2_]) also reduce grain quality. To determine whether heat-tolerant cultivars also tolerate E-[CO_2_], we conducted a free-air CO_2_ enrichment (FACE) experiment with 12 rice cultivars differing in heat tolerance.

**Results:**

The percentage of undamaged grains of five standard cultivars (Akitakomachi, Kinuhikari, Koshihikari, Matsuribare, Nipponbare) averaged 61.7% in the ambient [CO_2_] (AMB) plot and 51.7% in the FACE plot, whereas that of heat-tolerant cultivars (Eminokizuna, Wa2398, Kanto 257, Toyama 80, Mineharuka, Kanto 259, Saikai 290) averaged 73.5% in AMB and 71.3% in FACE. This resulted in a significant [CO_2_] by cultivar interaction. The percentage of white-base or white-back grains increased from 8.4% in AMB to 17.1% in FACE in the sensitive cultivars, but from only 2.1% in AMB to only 4.4% in FACE in the heat-tolerant cultivars.

**Conclusion:**

Heat-tolerant cultivars retained their grain appearance quality at E-[CO_2_] under present air temperatures. Further improvements in appearance quality under present conditions will be needed to achieve improvements under E-[CO_2_], because E-[CO_2_] will likely lower the threshold temperature for heat stress.

## Background

High temperatures during grain filling often cause serious damage to the grain quality of rice (*Oryza sativa* L.) (Terashima et al. [2001]; Lanning et al. [2011]; Kondo et al. [2012]), reducing the proportion of first-grade rice (Hasegawa et al. [2009]) or milling quality (Lyman et al. [2013]). One of the main reasons for the reduction is the decreased percentage of undamaged grains, which is due to increased proportions of chalky and unfilled grains. The occurrence of chalky grains under high temperatures is attributable mainly to the inhibition of starch accumulation (Morita [2008]; Morita and Nakano [2011]).

Daily mean temperatures >26°C during the grain-filling period cause chalkiness in the grains of *japonica* cultivars (Morita [2008]). The cumulative temperature above 26°C within 15 days after heading can be used as an index of the extent of chalky grains (Lur et al. [2009]). Daily mean air temperatures of >26°C during grain filling are becoming more frequent in Japan. Ishigooka et al. ([2011]) analyzed temperature records during the 20 days after heading in the Kanto, Hokuriku, Tokai, and Kinki regions from 1978 to 2010. Large areas in Tokai and Kinki experienced an increasing frequency of high temperatures, with low interannual variability. Kanto and Hokuriku experienced high interannual variability, with ≥80% of paddy fields under heat stress (1994, 2007, 2010 in Kanto; 1985, 1994, 2010 in Hokuriku).

Because grain quality under heat stress varies substantially among cultivars (Nishimura et al. [2000]; Wakamatsu et al. [2007]), improvement of heat tolerance is one of the most effective countermeasures (Morita [2008]). In fact, breeders have already developed some heat-tolerant cultivars in Japan, including ‘Nikomaru’ (Sakai et al. [2007]; [2010]), ‘Kumasannochikara’ (Fujii et al. [2009]), ‘Genkitsukushi’ (Wada et al. [2010]), and ‘Akisakari’ (Tanoi et al. [2010]), and there are a number of ongoing efforts to develop cultivars with improved heat tolerance.

The atmospheric CO_2_ concentration ([CO_2_]) is increasing. [CO_2_] has increased from 280 μmol mol^−1^ in 1800 to 396 μmol mol^−1^ in March 2013 (NOAA [2013]). Its continued increase is expected to have a big influence on crop production. Elevated [CO_2_] (E-[CO_2_]) is expected to increase the grain yield of rice (Kobayashi et al. [2006]; Hasegawa et al. [2007], Ainsworth [2008]), but it decreases the protein content (Lieffering et al. [2004]; Terao et al. [2005]; Yang et al. [2007]; Taub et al. [2008]; Seneweera [2011]) and the proportion of undamaged grains (Yang et al. [2007]). These studies suggest that production of high-quality rice grains will become even more difficult under the expected higher air temperatures and [CO_2_], but attempts to cope with both factors have yet to begin.

The mechanism of the reduction in undamaged grains under E-[CO_2_] is not understood, but a possible mechanism is a higher canopy temperature in E-[CO_2_] than in ambient [CO_2_] (AMB) conditions, which was observed in a free-air CO_2_ enrichment (FACE) study (Yoshimoto et al. [2005]). If higher canopy temperature due to E-[CO_2_] is the main reason for the reduced grain appearance quality, heat-tolerant cultivars might also be resistant to the occurrence of chalky grains under E-[CO_2_], but this hypothesis has never been tested. Therefore, we conducted a FACE experiment using several rice cultivars to determine whether heat-tolerant cultivars could maintain high grain quality under E-[CO_2_].

## Results

### Temperatures conditions and heading dates

The daily mean air temperature throughout the growing season in 2012 was 23.7°C, slightly higher than the 30-year average of 23.2°C obtained at the nearest weather station (AMeDAS at Tsukuba; 36°3.4′N, 140°7.5′E, 25 m above sea level). Heading date ranged from 24 July to 7 August, depending on cultivar (Table 1), but the daily mean air temperature averaged over the 20 days after heading (T_20DAH_), an important determinant of grain quality, varied only in a limited range from 26.5 to 26.9°C, which exceeded the threshold for the occurrence of chalky grains (Morita [2008]). Heading date was significantly earlier in the FACE plot than in the AMB plot (*P <* 0.05), but by only 1 day, and did not affect T_20DAH_ (Table 1).

**Table 1:** **Maturation, heading date, average temperature of T**_**20DAH**_**, tolerance rank, and notes for rice cultivars under test**

**Cultivargroup**^**A**^	**Cultivar(CV)**	**Heading date**		**T**_**20DAH**_^**B**^**°C**		**Tolerancerank**^**C**^	**Year ofrelease**	**Note**
		**FACE**	**AMB**	**FACE**	**AMB**			
ST	Akitakomachi	7/24	7/26	26.8 ± 0.3	26.9 ± 0.3	S	1982	Standard cultivar in this study
	Kinuhikari	7/30	7/30	26.7 ± 0.3	26.7 ± 0.3	S	1983	Big grains, Standard cultivar in this study
	Koshihikari	7/31	8/1	26.6 ± 0.3	26.5 ± 0.3	MT	1953	Standard cultivar in this study
	Nipponbare	8/5	8/6	26.6 ± 0.3	26.6 ± 0.3	MT	1961	Standard cultivar in this study
	Matsuribare	8/6	8/7	26.6 ± 0.3	26.6 ± 0.3	S	1990	Standard cultivar in this study
HT	Mineharuka	7/29	7/30	26.8 ± 0.3	26.7 ± 0.3	VT	2002	Long & thin grains (Saka et al., [2007])
	Toyama 80	7/30	7/31	26.7 ± 0.3	26.6 ± 0.3	VT	2012	*Apq1* on chromosome 7 (Ebitani et al., [2013])
	Eminokizuna	7/31	7/31	26.6 ± 0.3	26.6 ± 0.3	VT	2008	Small grains, useful for sushi
	Wa2398	7/31	7/31	26.6 ± 0.3	26.6 ± 0.3	VT	not released	QTL on chromosome 2. Derived from ‘Ma Li Xian’
	Kanto 257	8/1	8/1	26.5 ± 0.3	26.5 ± 0.3	T	2011	Derived from Mineharuka × Ikuhikari
	Kanto 259	8/5	8/5	26.6 ± 0.3	26.6 ± 0.3	MT– T	2012	High-yielding cultivar
	Saikai 290	8/6	8/6	26.6 ± 0.3	26.6 ± 0.3	VT	2012	High-yielding cultivar; good quality, good eating quality
Source of variation	df	Statistical significance						
Block	3							
CO_2_	1	*						
Main plot error	3							
CV	11	***						
Group^D^	1	*P =* 0.055						
ST^D^	4	***						
HT^D^	6	***						
CO_2_ × CV	11	*						
CO_2_ × Group^E^	1	ns						
CO_2_ × ST^E^	4	**						
CO_2_ × HT^E^	6	ns						
Split plot error	66							

^A^ST, standard cultivars; HT, heat-tolerant cultivars.

^B^T_20DAH_, air temperatures averaged for 20 days after heading ± standard error of mean.

^C^VT, very tolerant; T, tolerant; MT, moderately tolerant; S, susceptible.

^D^Sub-division of variation among the cultivars.

^E^Sub-division of variation among the CO_2_ × CV interaction.

***P < 0.001; **P < 0.01; *P < 0.05; ns, not significant.

### Grain appearance quality

The percentage of undamaged grains (UDG) was lower in FACE than in AMB by 5.4 percentage points (Table 2, *P =* 0.083), averaged over all cultivars, but with highly significant cultivar differences (*P* < 0.001). The reduction in UDG by E-[CO_2_] also differed significantly among cultivars, as evidenced by a highly significant [CO_2_] × cultivar interaction (*P* < 0.001; Table 2). This interaction was largely associated with the interaction with cultivar group (*P <* 0.001), whereas [CO_2_] × HT cultivars was not significant. Namely, the five standard (ST) cultivars (Akitakomachi, Koshihikari, Kinuhikari, Matsuribare, and Nipponbare) showed a large reduction of 10.0 percentage points (from 61.7% to 51.7%), whereas the heat-tolerant (HT) cultivars (Eminokizuna, Wa2398, Kanto 257, Toyama 80, Mineharuka, Kanto 259, Saikai 290) showed a reduction of only 2.2% (from 73.5% to 71.3%). The reduction in UDG was also different among the ST cultivars, as indicated by a significant [CO_2_] × ST cultivars interaction (*P <* 0.01, Table 2).

**Table 2:** **The effect of E-[CO**_**2**_**] on the percentage of grains categorized by appearance**

		**Grain appearance category**^**B**^									
**Cultivar group**^**A**^	**Cultivar (CV)**	**Undamaged**		**Cracked**		**Milky-white**		**Basal-white & White-Back**		**White-belly**	
		**(%)**		**(%)**		**(%)**		**(%)**		**(%)**	
		**FACE**	**AMB**	**FACE**	**AMB**	**FACE**	**AMB**	**FACE**	**AMB**	**FACE**	**AMB**
ST	Akitakomachi	40.2	58.0	1.6	1.8	5.7	4.8	28.9	10.8	4.3	2.3
	Kinuhikari	68.7	72.5	0.5	0.9	3.3	2.9	8.0	4.1	1.5	2.0
	Koshihikari	68.4	74.7	1.2	1.4	3.0	2.0	9.8	5.1	1.9	1.5
	Nipponbare	52.9	60.8	0.4	0.2	10.4	6.6	12.8	6.9	5.3	2.4
	Matsuribare	28.2	42.4	0.1	0.0	15.4	8.8	26.2	15.2	7.6	4.4
HT	Mineharuka	68.2	71.3	0.1	0.2	3.4	2.1	5.6	2.6	1.1	0.9
	Toyama 80	71.9	75.6	4.1	4.1	2.8	2.1	3.9	1.3	1.4	1.8
	Eminokizuna	85.0	85.7	0.1	0.0	1.6	0.7	1.1	0.7	0.8	0.5
	Wa2398	80.4	82.7	0.3	0.2	1.7	1.2	3.2	1.3	1.6	1.2
	Kanto 257	74.2	76.1	0.2	0.1	2.8	2.0	3.6	1.7	2.0	1.8
	Kanto 259	55.7	57.6	0.2	0.1	8.0	5.8	8.3	4.5	4.3	2.4
	Saikai 290	63.8	65.3	0.7	0.6	5.4	4.1	5.2	2.6	6.2	4.8
	ST mean	51.7	61.7	0.8	0.9	7.6	5.0	17.1	8.4	4.1	2.5
	HT mean	71.3	73.5	0.8	0.8	3.6	2.6	4.4	2.1	2.5	1.9
12 cultivars mean		63.1	68.5	0.8	0.8	5.3	3.6	9.7	4.7	3.2	2.2
Source of variation	df	Statistical significance									
Block	3										
CO_2_	1	*P* = 0.083		ns		*		*		*	
Main plot error	3										
CV^C^	11	***		***		***		***		***	
Group	1	***		*		***		***		***	
ST^C^	4	***		***		***		***		***	
HT^C^	6	***		***		***		***		***	
CO_2_ × CV	11	***		ns		*P* = 0.068		***		***	
CO_2_ × Group^D^	1	***		ns		ns		***		*	
CO_2_ × ST^D^	4	**		ns		**		***		***	
CO_2_ × HT^D^	6	ns		ns		ns		ns		ns	
Split plot error	66										

^A^ST, standard cultivars; HT, heat-tolerant cultivars.

^B^Percentage of grain number by appearance categorized by grain quality inspector.

^C^Sub-division of variation among the cultivars.

^D^Sub-division of variation among the CO_2_ × CV interaction.

***P < 0.001; **P < 0.01; *P < 0.05; ns, not significant; values indicate 0.05 < *P* < 0.1.

**Figure 1:**
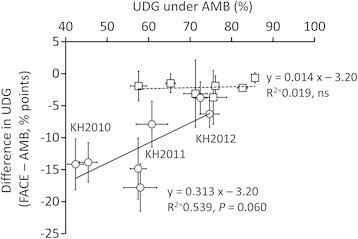
**Relationship between decrease in percentage undamaged grain (UDG) and increase in white-base and white-back grains (WBBG) by E-[CO**_**2**_**] among the 12 rice cultivars.**

The reduction in UDG was due mainly to an increase in the percentage of chalky grains (Figure 1), in particular, of white-back and white-base grains (WBBG: with a chalky area in the back of the grain or close to the basal part). WBBG increased from 8.4% in AMB to 17.1% in FACE in the ST group, but from only 2.1% to only 4.4% in the HT group (Table 2). This resulted in a highly significant [CO_2_] × cultivar group interaction (*P <* 0.001) and a non-significant [CO_2_] × HT cultivars interaction (Table 2).

Other types of chalky grains were also increased by E-[CO_2_]: the percentage of milky-white grains was 3.6% in AMB and 5.3% in FACE (*P <* 0.05, Table 2), and that of white-belly grains was 2.2% in AMB and 3.2% in FACE (*P <* 0.05), with highly significant cultivar differences (*P <* 0.001). The [CO_2_] × cultivar group interaction was significant for white-belly grains (*P* < 0.05), whereas the CO_2_ × HT cultivar interaction was not significant, but the magnitudes of the E-[CO_2_] effect and the interaction with cultivars were much smaller than in WBBG.

### Grain shape and weight

Averaged over all cultivars, E-[CO_2_] increased grain volume by 0.2 mm^3^ or 1.4% (*P* < 0.05, Table 3). Among the three components of grain volume, the effect of E-[CO_2_] was significant only for thickness (*P* < 0.001, Table 3), which accounted for most of the increase in volume due to E-[CO_2_]. The main effect of E-[CO_2_] on grain length was not significant, but there was a significant [CO_2_] × cultivar interaction (*P* < 0.01). There were also significant [CO_2_] × cultivar interactions for volume (*P* < 0.01) and thickness (*P* < 0.001), indicating that E-[CO_2_] had different effects on these traits, depending on cultivar. However, these traits are similar between the ST and HT groups among and across the CO_2_ treatments as evidenced by non-significant [CO_2_] × cultivar group interaction (Table 3) for any of the grain shape components.

**Table 3:** **The effect of E-[CO**_**2**_**] on shape, protein content, 1000-grain weight and brown rice yield**

		**Grain volume(mm**^**3**^**)**		**Grain shape**						**1000-grain weight**^**B**^**(g)**		**Gain protein content**^**B**^**(mg g**^**−1**^**)**		**Brown rice yield**^**B**^**(g m**^**−2**^**)**	
**Cultivar Group**^**A**^	**Cultivar (CV)**			**Length(mm)**		**Width(mm)**		**Thickness(mm)**							
		**FACE**	**AMB**	**FACE**	**AMB**	**FACE**	**AMB**	**FACE**	**AMB**	**FACE**	**AMB**	**FACE**	**AMB**	**FACE**	**AMB**
ST	Akitakomachi	14.3	14.2	5.08	5.08	2.71	2.71	1.99	1.98	22.0	22.2	63.5	63.9	581	567
	Kinuhikari	14.5	14.4	4.95	4.97	2.80	2.80	2.00	1.98	22.6	22.2	64.3	66.0	577	517
	Koshihikari	14.1	14.3	5.01	5.05	2.77	2.79	1.94	1.95	21.6	21.8	57.4	63.0	679	604
	Nipponbare	14.6	13.9	5.04	4.96	2.76	2.74	2.01	1.96	22.1	21.4	58.0	61.3	415	374
	Matsuribare	13.4	13.0	4.96	4.92	2.67	2.68	1.94	1.89	20.7	19.4	60.1	59.9	580	419
HT	Mineharuka	15.0	14.8	5.39	5.38	2.62	2.62	2.03	2.01	22.5	22.2	56.4	63.1	535	518
	Toyama 80	14.1	14.1	4.96	4.99	2.77	2.76	1.96	1.95	21.7	21.6	53.3	56.6	578	557
	Eminokizuna	14.0	13.9	5.01	5.02	2.64	2.65	2.02	2.00	21.4	21.8	74.9	72.5	535	496
	Wa2398	14.2	14.1	5.00	5.02	2.76	2.77	1.96	1.94	21.3	21.6	65.1	67.4	540	457
	Kanto 257	14.8	14.8	5.34	5.33	2.69	2.70	1.97	1.97	22.7	23.3	55.0	59.8	564	538
	Kanto 259	14.7	13.9	5.21	5.12	2.72	2.72	1.98	1.91	22.6	21.3	54.6	57.8	506	410
	Saikai 290	14.8	14.5	4.96	4.93	2.87	2.86	1.99	1.96	22.5	21.8	59.5	58.8	583	475
	ST mean	14.2	14.0	5.01	5.00	2.74	2.74	1.98	1.95	21.8	21.4	60.7	62.8	566	496
	HT mean	14.5	14.3	5.12	5.11	2.72	2.72	1.99	1.96	22.1	21.9	59.8	62.3	549	493
12 cultivars mean		14.4	14.2	5.08	5.06	2.73	2.73	1.98	1.96	22.0	21.7	60.2	62.5	556	494
Source of variation	df	Statistical significance													
Block	3														
CO_2_	1	*		ns		ns		***		*P =* 0.060		*P =* 0.083		*P =* 0.066	
Main plot error	3														
CV	11	***		***		***		***		***		***		***	
Group^C^	1	***		***		***		**		***		ns		ns	
ST^C^	4	***		***		***		***		***		***		***	
HT^C^	6	***		***		***		***		***		***		*P =* 0.053	
CO_2_ × CV	11	**		**		ns		***		***		**		ns	
CO_2_ × Group^D^	1	ns		ns		ns		ns		ns		ns		ns	
CO_2_ × ST^D^	4	**		**		ns		**		**		*P =* 0.095		ns	
CO_2_ × HT^D^	6	*		*		ns		**		***		**		ns	
Split plot error	66														

^A^ST, standard cultivars. HT, heat-tolerant cultivars.

^B^Expressed on a 15% moisture content basis.

^C^Sub-division of variation among the cultivars.

^D^Sub-division of variation among the CO_2_ × CV interaction.

***P < 0.001; **P < 0.01; *P < 0.05; ns, not significant. Values indicate 0.05 < *P* < 0.1.

Grain weight was also increased by E-[CO_2_] (*P =* 0.060, Table 3), with highly significant cultivar differences (*P <* 0.001) and a highly significant [CO_2_] × cultivar interaction (*P <* 0.001). In each cultivar group, there was also considerable variation in the response of grain weight to the CO_2_ treatment (*P <* 0.01 for the [CO_2_] × ST interaction and *P <* 0.001 for the [CO_2_] × HT interaction, Table 3), but the response was similar across cultivar groups.

### Grain protein content

The grain protein content (expressed on a 15% moisture content basis) averaged over the 12 cultivars dropped, from 62.5 mg g^−1^ in AMB to 60.2 mg g^−1^ in FACE (*P =* 0.083, Table 3). The effect of E-[CO_2_] differed significantly among cultivars, as evidenced by a [CO_2_] × cultivar interaction (*P* < 0.01), but the reduction in protein content by E-[CO_2_] was similar between ST and HT cultivars: from 62.8 mg g^−1^ in AMB to 60.7 mg g^−1^ in FACE in the ST cultivars, and from 62.3 mg g^−1^ in AMB to 59.8 mg g^−1^ in FACE in the HT cultivars (ns for the [CO_2_] × cultivar group interaction, Table 2).

### Yield

The average brown rice yield of all 12 cultivars increased from 494 g m^−2^ in AMB to 556 g m^−2^ in FACE (12.5%, *P =* 0.066). The yield enhancement in the two cultivar groups was similar—14.2% in ST and 11.3% in HT—and there was no [CO_2_] × cultivar interaction.

### Relationships among chalky grain percentage, protein content, and grain shape

Multiple-regression analysis showed that grain shape (length, width, and thickness), weight, and protein content were not associated with cultivar differences in the UDG response to E-[CO_2_] (data not shown).

## Discussion

E-[CO_2_] significantly reduced grain appearance quality by increasing the percentage of chalky grains, as was reported in a previous FACE experiment in China (Yang et al. [2007]). Here, we showed that the effect of E-[CO_2_] on grain appearance differed widely among cultivars, and that newly developed heat-tolerant cultivars retained high quality under E-[CO_2_]. These results suggest that the current efforts in breeding for heat tolerance will also be effective under projected climate change.

The mechanism by which the grain appearance quality is reduced by E-[CO_2_] is not yet understood. E-[CO_2_] might be expected to improve appearance quality because of higher rates of photosynthesis and of assimilate supply to the grains. In fact, a growth chamber study revealed a modest but positive effect of E-[CO_2_] (Ward [2007]). However, this advantage did not appear under the FACE conditions, likely because of various indirect effects of E-[CO_2_] on the physiology of the crop grown in open field conditions.

Among various types of chalky grains that degrade grain appearance quality, we observed significant increases in the percentage of WBBG at E-[CO_2_] (Table 2), which is known to increase under high temperatures and limited N (Kondo et al. [2006]; Wakamatsu et al. [2007]). We applied the same amount of N fertilizers to all cultivars under both [CO_2_] conditions, but E-[CO_2_] decreased grain protein content (Table 3), as observed in many other studies (Lieffering et al. [2004]; Terao et al. [2005]; Yang et al. [2007]; Taub et al. [2008]; Seneweera [2011]; Zhang et al. [2013]). The reason for the different responses in the occurrences of chalky grain to E-[CO_2_] among cultivars is not clear. If N-related processes are involved in the occurrence of E-[CO_2_] induced chalkiness, one can hypothesize that cultivars with a smaller loss in grain protein content under E-[CO_2_] may show a smaller reduction in grain quality. However, we did not observe noticeable differences in the grain protein response to E-[CO_2_] between ST and HT groups. Some HT cultivars with a small reduction of grain quality also showed a slightly larger reduction of protein content than ST cultivars. This suggests that the quantity of protein does not have direct relevance to the response of appearance quality to E-[CO_2_]. While we need further studies to determine whether the quantity or activity of specific proteins or enzymes plays a role in the response, the rates of chalkiness could be reduced even with a lower protein content, which is highly important for eating quality.

E-[CO_2_] reduces leaf stomatal conductance, which is commonly observed across many plant species (reviewed by Ainsworth and Rogers [2007]). This could result in a higher canopy temperature under E-[CO_2_] than under ambient [CO_2_] because of reduced transpirational cooling. We did not measure canopy or panicle temperatures in this study, but a previous rice FACE study reported a 0.2 to 1°C increase in canopy temperature by E-[CO_2_] (Yoshimoto et al. [2005]), which likely occurred in our FACE experiment. Some recent studies highlighted the importance of canopy temperature rather than air temperature in heat stress (Matsui et al. [2007]; Julia and Dingkuhn [2013]; Zhao and Fitzgerald [2013]). These results suggest that E-[CO_2_] induces chalky grains through increased canopy or panicle temperature. Evidence also exists that canopy temperatures vary significantly among genotypes (Takai et al. [2010]), so the difference in foliage temperatures may partially account for the cultivar differences in the response to E-[CO_2_]. The canopy microclimate of different cultivars could also be an important subject for future study.

**Figure 2:**
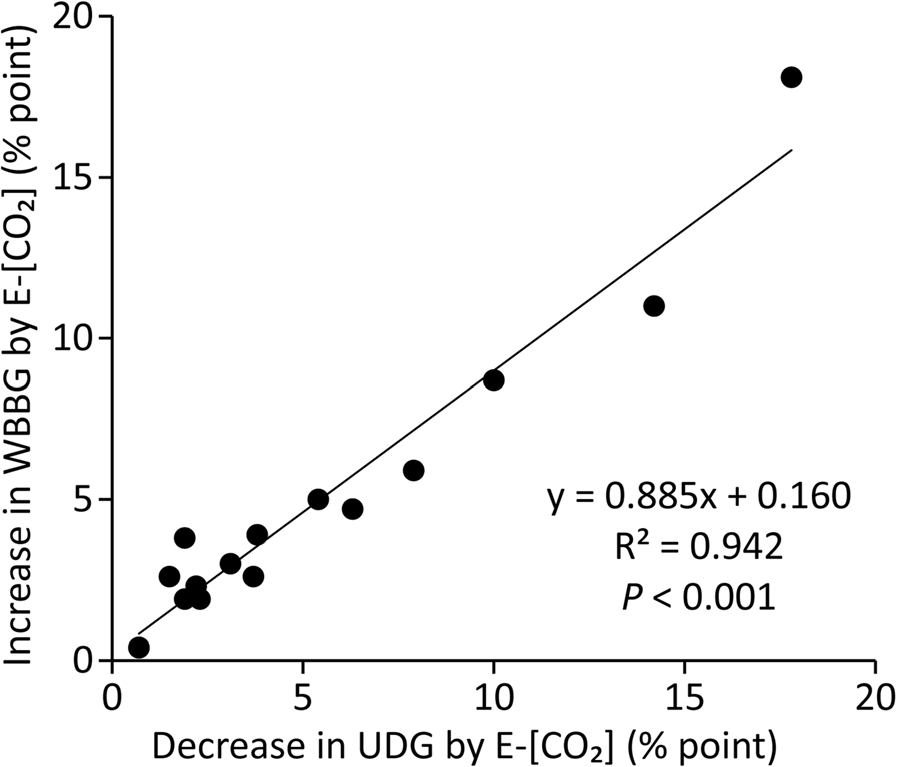
**Relationship between the percentage of undamaged grains (UDG) in AMB and difference in UDG between FACE and AMB.** KH = Koshihikari (tested in 2010–2012). ○, Standard cultivar; □, Heat-tolerant cultivar. Error bars represent standard error of the mean (*n* = 4).

Grain appearance quality often varies from year to year with seasonal and weather conditions. The loss of grain quality due to hot summers has been reported recently both in Japan and elsewhere (e.g., Kondo et al. [2012]; Lyman et al. [2013]). At the Tsukuba FACE site, we tested Koshihikari in 2010 and 2011 also, when the temperatures during the grain-filling period were higher than in 2012. As expected, UDG in AMB in these two years was smaller than in 2012. When we combined these two additional data points with those of the ST group in the 2012 season, the difference in UDG between FACE and AMB became larger as UDG in AMB decreased (Figure 2). This suggests that the negative effect of E-[CO_2_] on UDG can be exacerbated in hotter summers. In the HT group, the same relationship was not significant, suggesting that these cultivars are likely to show a smaller loss of grain quality under E-[CO_2_], but this needs to be tested under harsher environments.

E-[CO_2_] induced WBBG-type chalkiness, which has long been known as a result of high sensitivity to heat (Nagato and Ebata [1965]). All of the HT cultivars showed good appearance quality under both [CO_2_] conditions, indicating that selection for heat tolerance can be effective for improving grain appearance quality under E-[CO_2_]. The recent identification of a number of QTLs for heat-induced increases in white-back or white-base chalkiness (e.g., Kobayashi et al. [2007]; [2013]; Ebitani et al. [2008]) will support marker-assisted selection for high appearance quality under high temperatures and possibly under E-[CO_2_]. However, some of these QTLs may not be effective above a certain threshold; for instance, a T_20DAH_ of 27°C (Kobayashi et al. [2007]). Our results suggest that projected rises in [CO_2_] will lower the threshold for heat-induced chalkiness, so continued efforts will be needed to increase the level of heat tolerance. In the meantime, we need to improve our mechanistic understanding of how heat-tolerant cultivars could be buffered against loss of grain quality under elevated [CO_2_] in order to identify promising traits for future breeding programs.

Under the projected future climate change, both high productivity and high quality will be needed. In our previous study, we suggested the potential for improving productivity under E-[CO_2_] by showing cultivar differences of 3% to 36% in grain yield responses to E-[CO_2_] (Hasegawa et al. [2013]). Here, we did not observe a significant interaction in grain yield between [CO_2_] and cultivars, but neither did we find a negative relationship between quality and grain yield (r^2^ = 0.0281, ns). This suggests that both productivity and quality traits can be improved separately or simultaneously, but continued efforts are needed.

## Conclusions

These results indicate that heat-tolerant cultivars retain their grain quality under E-[CO_2_] better than standard cultivars. Thus, an improvement in grain quality under present conditions will achieve an improvement under E-[CO_2_]. However, continued efforts will be needed to improve heat tolerance, because E-[CO_2_] will likely decrease the threshold temperature for heat stress.

## Methods

### Study site

We conducted the FACE experiment at the Tsukuba FACE site, which was established in farmers’ fields in Tsukubamirai City, Ibaraki Prefecture, Japan (35°58′N, 139°60′E, 10 m above sea level). The climate is humid subtropical with an average annual temperature of 13.8°C and annual precipitation of 1280 mm. The soil is a Fluvisol, which is typical of alluvial areas. The soil properties at the site are described by Hasegawa et al. ([2013]); in brief, the soil contains 21.4 mg g^−1^ total C and 1.97 mg g^−1^ total N, has a bulk density of 0.87 Mg m^−3^, and has a composition of 36% sand, 40% silt, and 23% clay.

### CO_2_ treatment

The method for controlling [CO_2_] in an open field is described by Nakamura et al. ([2012]). In brief, four blocks (replicates) were established in paddy fields, each consisting of two octagonal plots (240 m^2^, 17 m across): an ambient [CO_2_] (AMB) plot and an E-[CO_2_] (FACE) treatment plot. The FACE plots were equipped with emission tubes around the perimeter, which released CO_2_ from the windward side to keep the [CO_2_] measured at the central point at ca. 200 μmol mol^−1^ above the AMB level. The season-long daytime average [CO_2_] in 2012 was 392 μmol mol^−1^ in the AMB plots and 577 μmol mol^−1^ in the FACE plots.

### Cultural practices and growth conditions

We applied compound fertilizer to supply 4.36 g m^−2^ of phosphorus (P) and 8.30 g m^−2^ of potassium (K) on 9 April 2012, before plowing. We applied a total of 8 g m^−2^ of nitrogen (N) fertilizer: 2 g m^−2^ as urea, 4 g m^−2^ as one controlled-release fertilizer (type LP100), and 2 g m^−2^ as another controlled-release urea (type LP140, JCAM Agri. Co., Tokyo, Japan). Right after N application, we puddled (tilled) the field for uniformity on 17 May in 2012.

In each plot, we planted 7 heat-tolerant cultivars and 5 standard cultivars (Table 1). Seedlings were transplanted by hand on 23–25 May 2012 at a spacing of 30 cm × 15 cm (22.2 hills m^−2^). Seedlings of Akitakomachi and Koshihikari, which we sampled during the growing season for other studies, were planted in areas of 2.7 m × 1.95 m (Akitakomachi) and 3 m × 5.4 m (Koshihikari) at three seedlings per hill. The others were planted in areas of 1.2 m × 0.45 m (12 hills) per cultivar in each plot; they were randomly allocated within the plot.

### Measurement

We sampled 21 hills of Akitakomachi and Koshihikari and 12 hills of the others (11 of Wa2398 in one FACE plot) in each of the four replicates for grain yield components and quality measurement. After the materials were dried under a rain shelter, we measured the total aboveground plant weight and panicle number. After threshing, we measured the total weight of the spikelets. Each spikelet sample was then split into three subsamples. One subsample was dehulled to determine the brown rice weight; this sample was then used for grain quality measurement in this study. The other 2 subsamples were used in another study. We measured the moisture content of the grains with a grain moisture tester (Riceter f, Kett Electric Laboratory, Tokyo, Japan). The brown rice yield and 1000-grain weight were expressed on a 15% moisture content basis.

We measured grain appearance quality (different types of chalky grains) and grain shape (width, length, thickness) with a grain quality inspector (RGQI20A; Satake Corp., Hiroshima, Japan) equipped with image analysis software. White-back and white-base grains were counted together, and are reported as WBBG. The grain volume was calculated as for an ellipsoidal body: 4/3 × π × length/2 × width/2 × thickness/2. N was measured with an NC analyzer (Sumigraph NC-22 F; Sumica Chemical Analysis Service, Tokyo, Japan). The protein content (%) was calculated as N (%) × 5.95 (MEXT [2005]) and corrected to 15% moisture content.

### Statistics

We conducted an analysis of variance for the 12 cultivars, using a split-plot design, where [CO_2_] was treated as the main factor and cultivar as the split factor, with four replications, in the SAS software, using the GLM procedure (SAS Institute Inc., Cary, NC, USA). To test differences between cultivar groups, we separated the sums of squares for the split-plot components into the following sources of variation; between-cultivar groups, within HT and ST groups and their interactions with [CO_2_] (Tables 1, 2, 3). To examine whether cultivar differences in the UDG response to E-[CO_2_] were associated with grain shapes (length, width, thickness), grain weight and grain protein content, we conducted multiple regression of the increase in UDG by elevated [CO_2_] on the log-transformed response ratio (FACE/ambient) of grain shape parameters. Statistical significance is indicated for *P* < 0.001, 0.01, or 0.05, and actual *P* values are presented where 0.05 < *P* < 0.1.

## Abbreviations

AMB: Ambient: 

FACE: Free-air CO_2_ enrichment: 

HT: Heat-tolerant cultivars: 

ST: Standard cultivars: 

T_20DAH_: Air temperatures averaged for 20 days after heading: 

UDG: Percentage of non-damaged grains: 

WBBG: White-based and white-back grains: 

## Competing interests

The authors declare that they have no competing interests.

## Authors’ contributions

TH planned and supervised the project. HS and TT designed the experimental plot. YU, HS, TT, HN, and TH conducted the FACE experiment. HN managed the [CO_2_] treatments and monitored the environmental variables. YU collected and analyzed the data. HN advised on grain quality measurements and interpretation of the results. YU and TH wrote the paper. All authors read and approved the final manuscript.
